# *Cor-Esc-25*: A Low-Cost Prototype for Monitoring Brace Adherence and Pressure in Adolescent Idiopathic Scoliosis

**DOI:** 10.3390/s25185616

**Published:** 2025-09-09

**Authors:** Pablo Ulldemolins, Pedro Rubio, Jorge Morales, Silvia Pérez, Jose Luis Bas, Paloma Bas, Mario Lamas, Jose María Baydal, Miquel Bovea, Carlos María Atienza, Teresa Bas

**Affiliations:** 1 Spine Unit, La Fe University and Polytechnic Hospital of Valencia, Avinguda de Fernando Abril Martorell, 106, Quatre Carreres, 46026 Valencia, Spain; 2 Institute of Biomechanics of Valencia, Camí de Vera, s/n, Algirós, 46022 Valencia, Spain

**Keywords:** open prototype, adolescent idiopathic scoliosis, pressure sensor, temperature sensor, smart brace

## Abstract

**Highlights:**

**What are the main findings?**

- We propose a novel, economical, and practical open-system prototype, which we have named *Cor-Esc-25*, to measure brace adherence and adjustment in adolescents with idiopathic scoliosis.
- Comparative analysis of the available non-invasive temperature and pressure sensors and creation of an organic system adaptable for any type of brace, which could help future researchers to understand the effectiveness of adherence and pressure in the correction of a Cobb curve.

**What is the implication of the main findings?**

- This study addresses a critical gap by establishing a universal, cost-effective prototype that provides researchers with objective data to study brace prescription effects.
- Its ease of application across different types of braces and low cost make *Cor-Esc-25* a highly attractive solution for resource-constrained environments, and a promising foundation for future research.

**Abstract:**

The treatment of adolescent idiopathic scoliosis (AIS) requires the use of orthopedic braces. However, few current designs provide real-time monitoring or inform clinicians about the precise adjustment of therapeutic pressure. The objective of this study is to develop a low-cost open-system prototype capable of providing future researchers with objective information regarding brace adherence and adjustment. For adherence evaluation, a market study was conducted to identify temperature-measuring devices and a custom system was developed to measure adjustment. *Cor-Esc-25* was developed to monitor brace adherence using a non-invasive temperature sensor which connects via Bluetooth to the parents’ smartphone, which runs an app that uploads the data to an online platform accessible to clinicians. In addition, a custom-designed pressure sensing device was created. This system uses three patches connected to an acquisition board and are installed on the brace each time the patient visits the clinic. It connects to a customized application where clinicians can view all the information. *Cor-Esc-25* represents a first step toward the creation of personalized consultations, where AIS treatment monitoring is based on objective criteria that consider both adherence and brace adjustment. Its design allows for easy integration into clinical settings, thereby improving the ability of researchers and clinicians to assess the effectiveness of brace treatment.

## 1. Introduction

The prevalence of adolescent idiopathic scoliosis (AIS) is 1–3% among the population aged 10–16 years [1]. According to Konieczny et al., 0.5–5.2% of the global population is affected by this condition [2]. The Cobb angle is used for diagnosis and follow-up, and curves between 20 and 45° are generally treated with thoracolumbar braces such as the Boston, Chêneau, or Milwaukee brace, with the goal of stabilizing the spine and preventing progression of the curvature [2]. If the Cobb angle increases, AIS can lead to long-term cardiopulmonary complications [3]. The effectiveness of bracing relies on two parameters: (1) adherence, or the duration of brace wear, and (2) efficacy, or the pressure applied at support points [4].

Wearing the brace 24 h a day has not been shown to yield significantly better outcomes, whereas adherence of less than 12 h per day appears insufficient. As a result, the current recommendation is to prescribe the brace for at least 18 h per day [4,5]. Measuring adherence based on the patient’s own reporting is subjective, prone to bias, and difficult to quantify. To overcome these limitations, tools are being developed to objectively measure adherence [5].

To estimate brace adherence, previous studies have used pressure threshold changes. However, pressure readings can drop to zero during self-correction exercises or due to a loose fit of the orthosis [6,7,8,9,10]. Therefore, pressure-based monitoring has limitations that may lead to an underestimation of actual wear time [6,7]. These limitations highlight the need for more comprehensive adherence monitoring methods. Among the options, thermal sensors capable of detecting body temperature have become a widely adopted solution [5,11,12].

Regarding the force the brace must exert, there is currently no consensus on the magnitude of corrective force required to achieve optimal therapeutic effects. Authors suggests that the necessary brace stiffness to produce beneficial effects could be achieved through simple tightening of Velcro straps, but these values are subjective and difficult to quantify [13]. Today, the available options for measuring brace pressure forces exceed the cost expected for a device accessible to most patients [8,14].

In this context, there is a need for accessible, integrated monitoring tools capable of capturing both adherence and pressure data in real-world conditions. The purpose of this article is therefore to present the design and preliminary bench testing of *Cor-Esc-25*, a low-cost prototype adaptable to standard braces, intended to quantify brace wear time and pressure at clinician-selected support points. This proof-of-concept aims to provide the basis for future validation in patients with AIS.

## 2. Materials and Methods

This study involved the design and creation of the *Cor-Esc-25* orthotic monitoring device, along with a pilot clinical trial with 4 patients to evaluate brace adherence and fit in patients with adolescent idiopathic scoliosis. The project was supported by a *EUR 6000* GEER 2022 grant.

### 2.1. Brace Selection

The protocol of the Pediatric Spine Unit at our center was followed for the treatment of AIS in patients with mild to moderate curves. Therefore, the brace selected for the development of the final device corresponds to a Boston-type brace, consisting of an outer shell made of copolymer polypropylene and an inner lining of self-adjusting polyethylene foam with Velcro straps. The *Cor-Esc-25* was installed in Boston braces prescribed to AIS patients during routine clinical practice, under the supervision of the treating physician.

### 2.2. Sensor Selection

To assess adherence, the proposed method involves using temperature sensors placed inside the brace. To measure pressure, force sensors were applied to the support points selected by the clinician.

A market study was conducted to select both types of sensors among commercially available options. Evaluation criteria prioritized the cost, availability in our environment, biocompatibility, and safety of the sensor. Among those that meet these requirements, priority was given to smaller devices with better precision, autonomy, connectivity, and ease of integration.

Although these sensors could present limitations (thermal lag in warm environments, drift and hysteresis in force-sensing resistors), they were considered suitable for proof-of-concept testing.

If no available options met the pre-established criteria, a custom-designed sensor was considered.

### 2.3. Sensor Calibration

Pressure sensors, FSR 01BE^®^ (Nanjing Momao Electronic Technology Co., Ltd., Nanjing, China), were calibrated using incremental loads applied on a compression bench. Output voltages were recorded and a polynomial regression was applied for linearization. Calibration was repeated in three cycles to assess repeatability. Temperature sensors, Tucky^®^ (e-TakesCare SAS, Versailles, France), were verified against a digital thermometer at room and body temperature (37 °C). A 30 °C threshold was predefined to classify brace wear.

### 2.4. Preliminary Design of Cor-Esc-25

To measure patient adherence, the temperature sensor must be integrated into the brace in such a way that (1) it remains continuously connected to detect when the patient is wearing the brace or not, and (2) the measurements do not interfere with the brace’s therapeutic effect. The selected or custom-made sensor must be small enough to be housed within the brace shell or inserted into one of the holes designed for ventilation.

**Figure 1:**
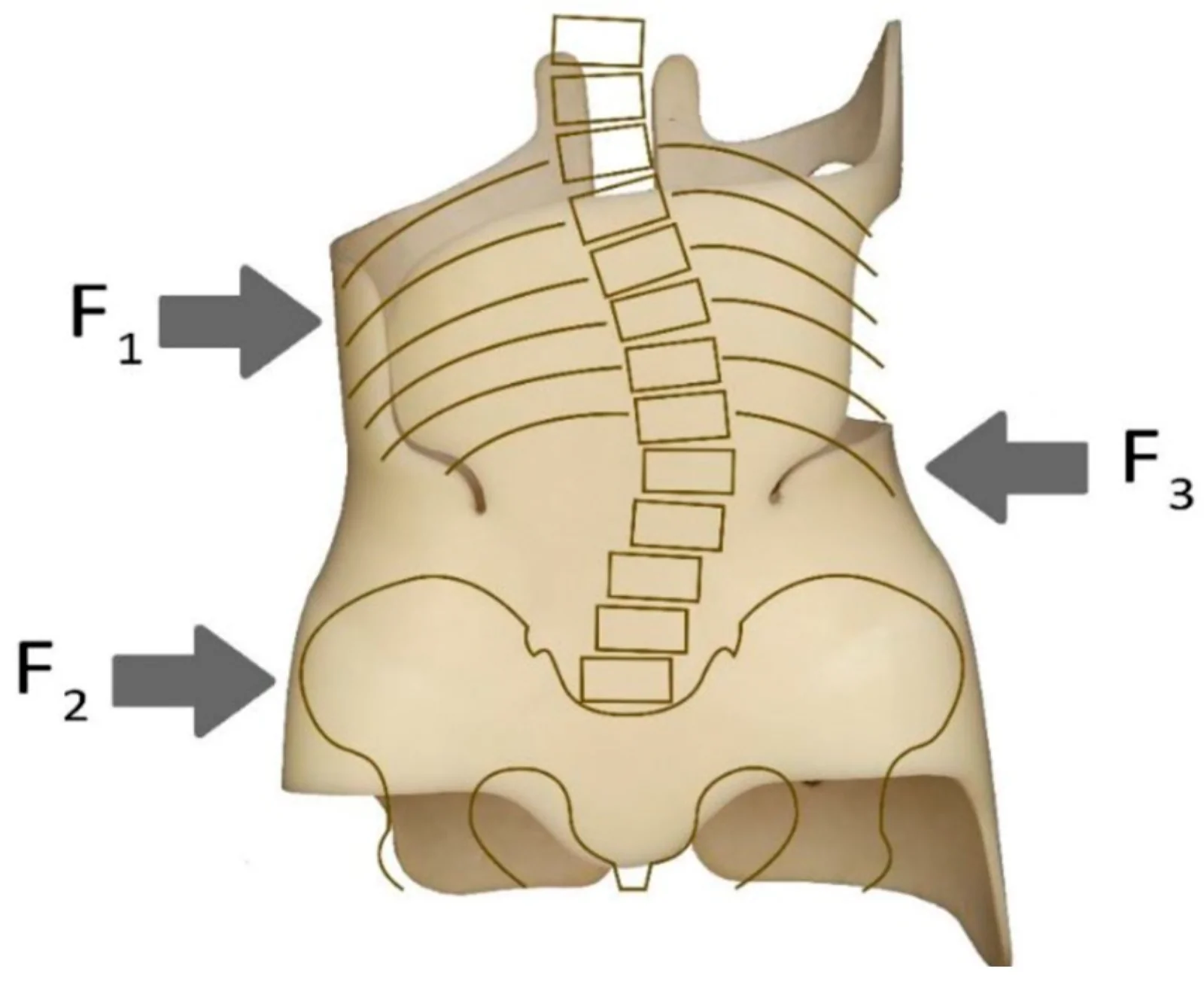
Representative diagram of the effect of the Boston brace with sensors placed at pressure points.

For measuring brace fit, pressure sensors were used to quantify the corrective forces exerted by the brace. As shown in Figure 1, the force distribution followed the scheme below:

- Sensor 1 [Figure 1—F1]: Pressure point on the convexity of the thoracic curve.
- Sensor 2 [Figure 1—F2]: Pressure point on the convexity of the lumbar curve.
- Sensor 3 [Figure 1—F3]: Located in the pelvic region.

For preliminary analysis, mean pressure values were calculated at each site. Wear time was estimated by setting a temperature threshold of 30 °C, above which the brace was considered “in use”.

### 2.5. Patient Testing

After bench calibration, *Cor-Esc-25* was tested in a small pilot series of 4 AIS patients wearing Boston braces. Inclusion criteria were AIS diagnosis, prescription of a Boston-type brace, and consent for temporary sensor placement. Pressure sensors (FSR 01BE^®^) were positioned at the thoracic, lumbar, and pelvic corrective points selected by the treating clinician.

Each patient wore the brace for 30 min in a seated and standing position. For each site, mean pressure, standard deviation, and peak values were obtained. This study was approved by the institutional ethics committee.

## 3. Results

### 3.1. Temperature Sensor

We conducted an exhaustive review of the different sensors available on the market that could be acquired at low cost. The evaluation prioritized characteristics such as biocompatibility, precision, autonomy, connectivity, ease of integration into a pre-designed brace, and cost.

Six potential temperature sensors met these criteria:

- ScolioSense Temperature Sensor^®^: A lightweight, discreet sensor specifically designed to integrate into the Boston brace. It provides effective monitoring of adherence to AIS treatment and includes a proprietary mobile app. Battery lasts 21 days. Price not available [15].
- Orthotimer^®^: A very compact thermal sensor (13 × 9 × 5 mm) for documenting adherence. It offers 400 days of storage, customizable recording intervals (from 1 s to 60 min), USB reader, Bluetooth data transfer, and cloud-based analysis software. Price: *EUR 2830* [16].
- Boston Sensor^®^**:** A wireless device placed inside the brace that records internal temperature every 15 min. About the size of a 50-cent coin, it includes an app (iOS/Android) and generates usage reports easily. One-year battery, replaceable with a standard watch battery. Compatible only with its dedicated app for privacy. Price not available [17].
- Core^®^: A clinically validated, portable, non-invasive device for continuous core temperature monitoring. Features ANT+ and Bluetooth connectivity, waterproof, compact design, and a rechargeable battery lasting up to 6 days. Price: USD 298.95 [18].
- Radius T^®^: A wireless, portable sensor that continuously measures body temperature every minute. Includes Bluetooth connectivity, water-resistant reusable adhesive, and a battery life of 8 days. Single-patient use. Indicated for patients aged 5 and older. Price: USD 49.99 [19].
- Tucky^®^: A thin, flexible, biocompatible thermometer in patch form developed by e-TakesCare, intended for non-invasive temperature monitoring under the armpit in preschool children. Transmits data via Bluetooth to a free smartphone app (iOS/Android), with real-time cloud syncing accessible to caregivers and clinicians. ISO13485 certified. Market price: ~EUR 70 [20].

**Figure 2:**
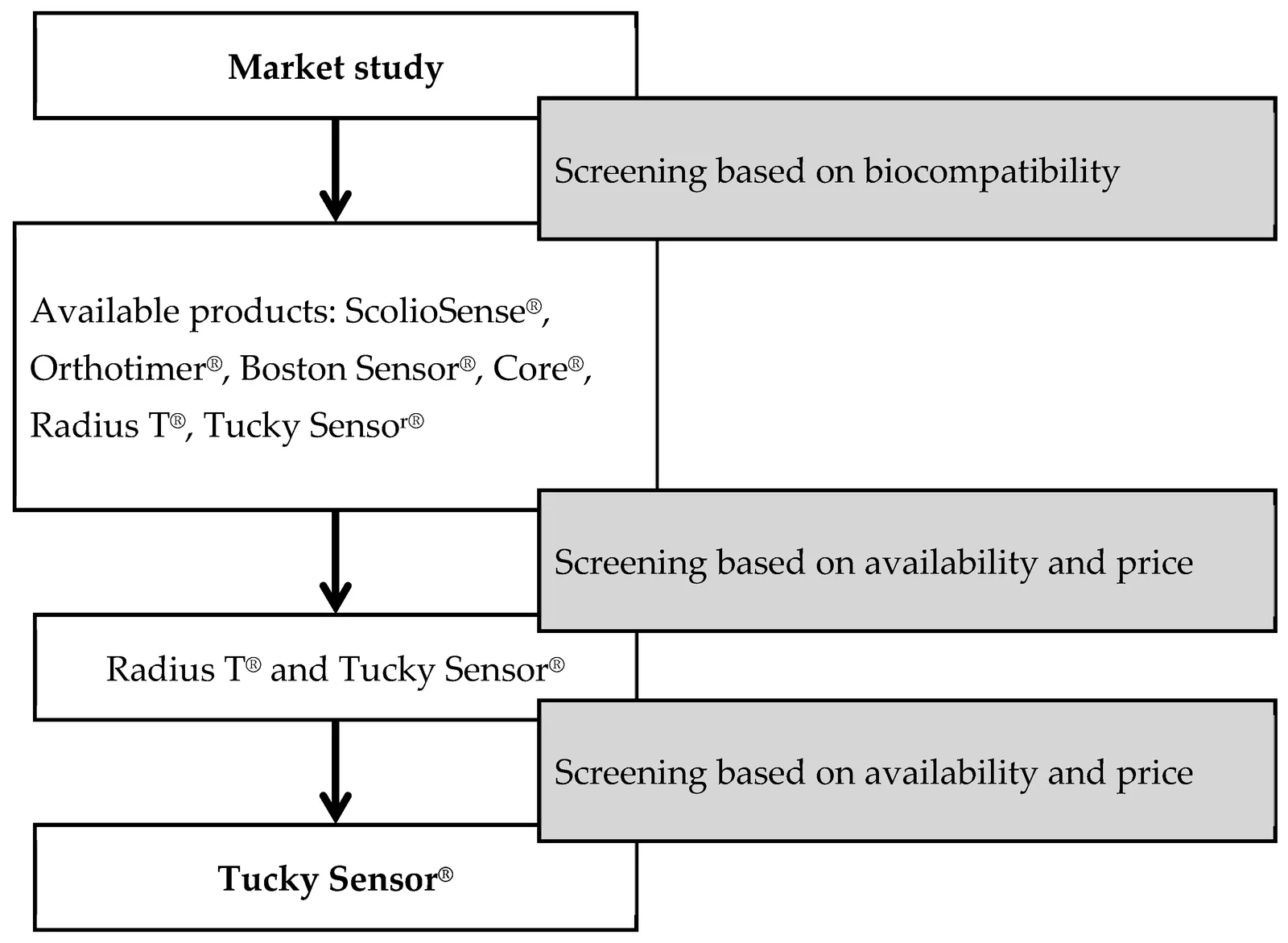
Decision tree for the selection of the temperature sensor.

Following the decision tree outlined in the methodology, the Tucky^®^ device was selected, as shown in Figure 2. A qualitative summary of the sensors selected in the market study is shown in Table 1.

### 3.2. Pressure Sensor

We also analyzed currently available options for pressure measurement:

- ScolioSense Pressure Sensor^®^: Designed to ensure effective AIS treatment by tracking brace wear time. This lightweight and discreet device integrates easily into the brace and connects to a mobile app. It provides real-time data on treatment progress. Battery lasts 21 days. Price quote not obtained [15].
- F-Socket Tucky^®^: Used to evaluate and monitor the effectiveness of orthotic braces in AIS treatment. Sensors are placed inside the brace, offering real-time pressure data for immediate adjustments. Data can be stored and analyzed over time. Price: EUR 25,549 [21].
- Spondylos Tucky^®^: Measures, records, and analyzes the forces exerted by a Boston-type brace. Portable and lightweight, it includes a microprocessor, microSD card, and warning LEDs that activate when pressure drops below a set threshold. Price not available [22].

As none of the options met the research team’s predefined criteria, we opted to design a custom system based on FSR01BE^®^ technology, priced between *EUR 5* and *EUR 6* [23].

**Figure 3:**
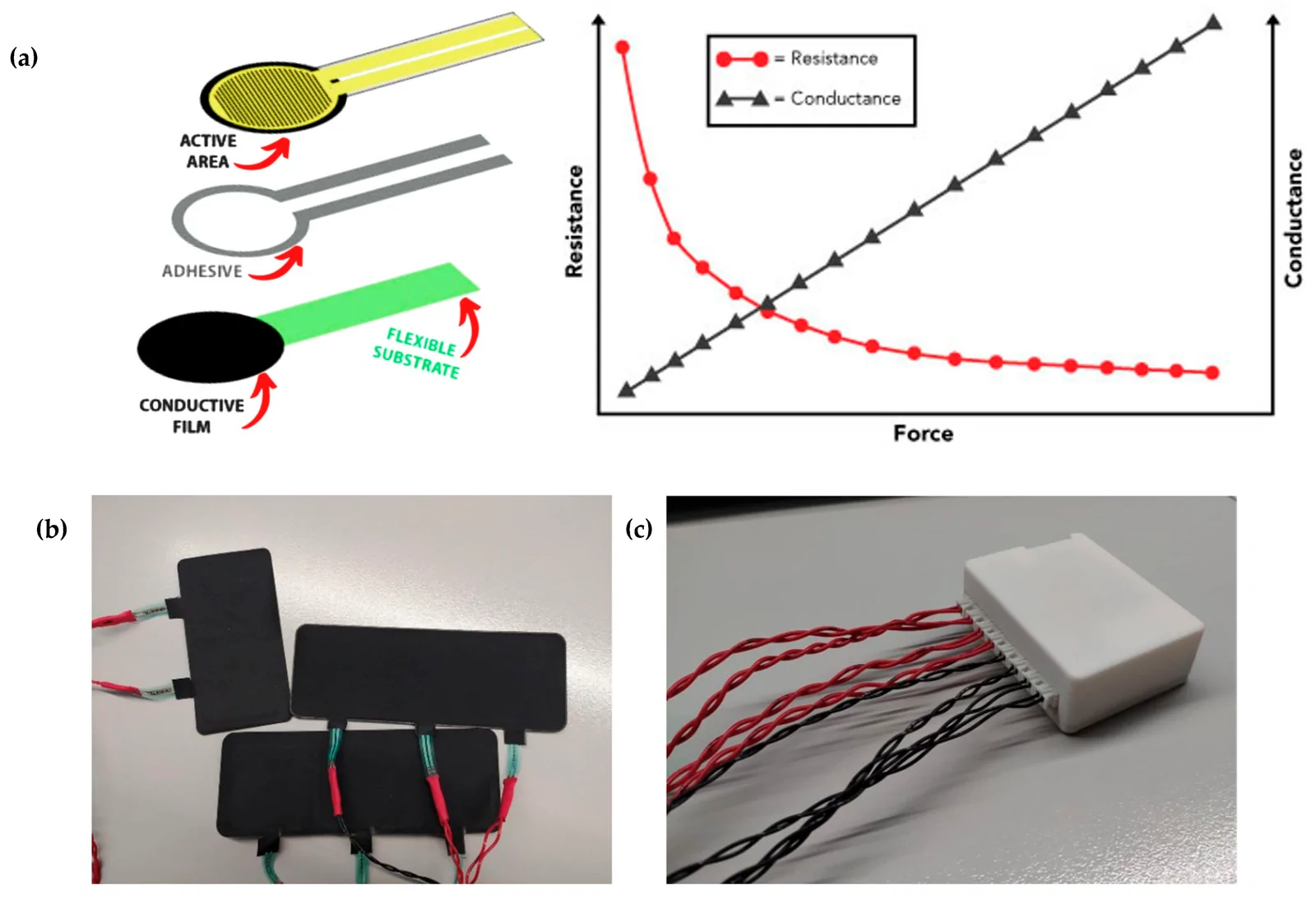
(**a**) Diagram of the FS401BE® sensor operation; (**b**) pressure sensor using FSR01BE^®^ technology (**c**) with the acquisition board.

The FSR01BE^®^ is a force-sensing resistor measuring 83.09 × 43.69 × 43.69 mm, with two pin outputs and an activation force of 20 g [23]. In general, the greater the applied force, the lower the sensor’s resistance (see Figure 3a). A custom acquisition board was designed to collect real-time resistance changes, as shown in Figure 3b,c.

FSR sensors were calibrated using incremental loads applied through a compression test bench. Output voltage was recorded, and a polynomial regression was fitted for linearization. Calibration was repeated three times to evaluate repeatability. The main limitations considered were drift over time, hysteresis during load/unload cycles, and variability depending on placement.

### 3.3. Cor-Esc-25 Design

**Figure 4:**
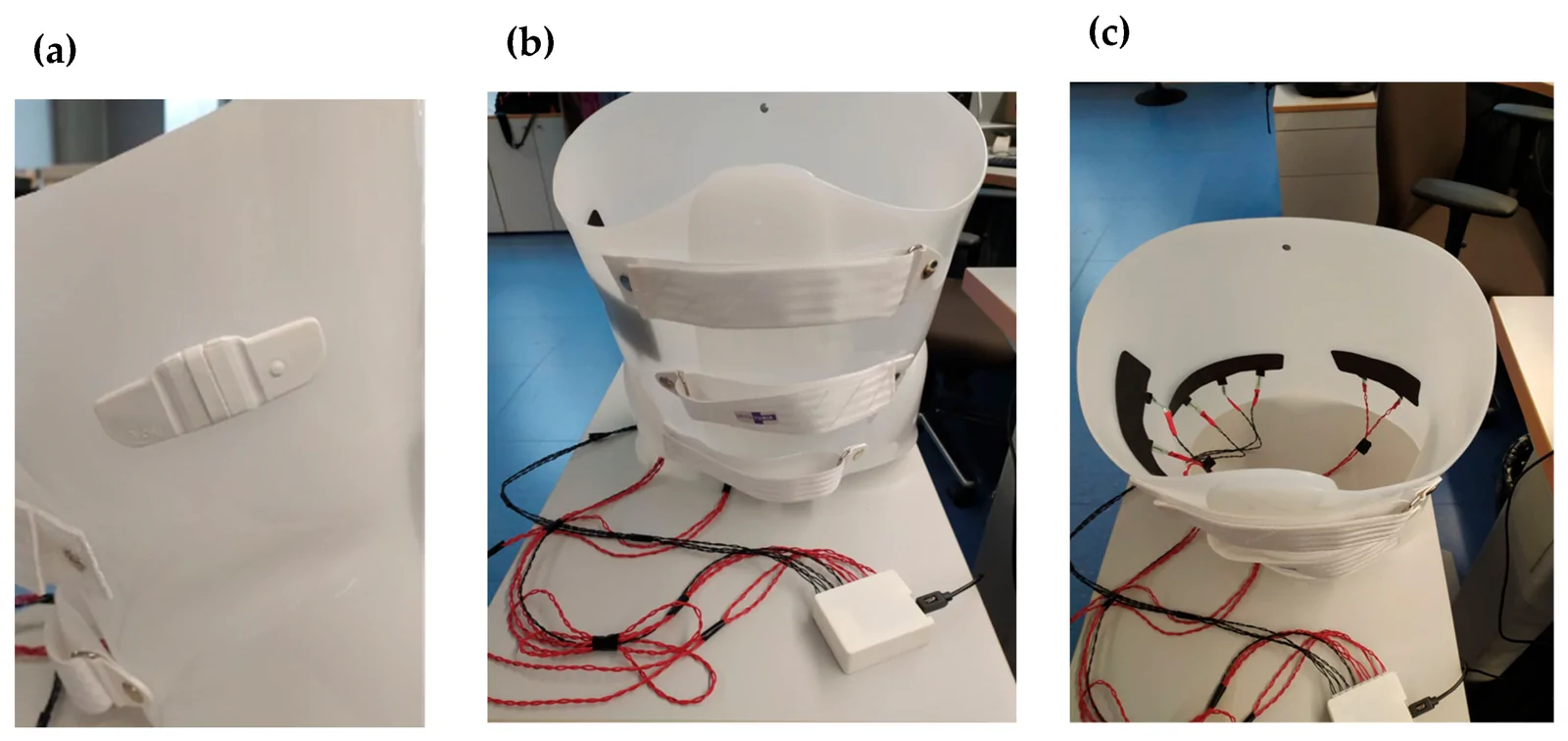
Images of the *Cor-Esc-25*. (**a**) Temperature sensor (Tucky^®^) inserted in a ventilation hole. (**b**) Anterior view of the brace with a custom acquisition board. (**c**) Pressure sensor inserted inside the brace.

The temperature sensor was attached to the standard brace using one of the built-in ventilation holes (Figure 4a). From this position, it can connect via Bluetooth to a nearby mobile device for continuous temperature logging.

As described in the methodology, three pressure points were selected: one at the apex of the thoracic curve, one at the lumbar curve, and one at the pelvis. This layout was designed to measure the corrective forces and to ensure a proper fit of the brace.

Due to its size, the system could not be organically integrated into a standard Boston brace. Instead, the system was temporarily installed during each check-up to assess brace adjustment. The selected pressure points were marked on the brace to ensure accurate, repeatable measurements. The sensors were covered with PSP Dacron11 adhesive tape to protect them without compromising sensitivity (Figure 4b,c).

### 3.4. Data Reading

**Figure 5:**
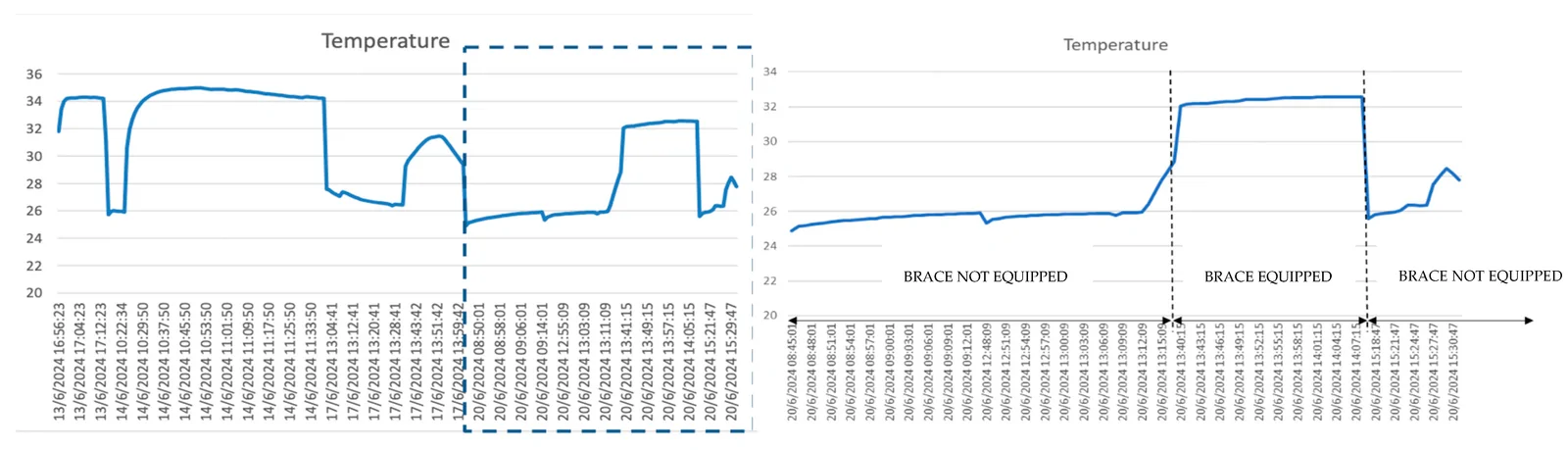
Images obtained from the Tucky^®^ application. If the sensor is connected to the mobile device via Bluetooth, it can continuously capture the temperature and upload it to the cloud. The moments when the brace is being worn and when it is removed can be clearly identified.

**Figure 6:**
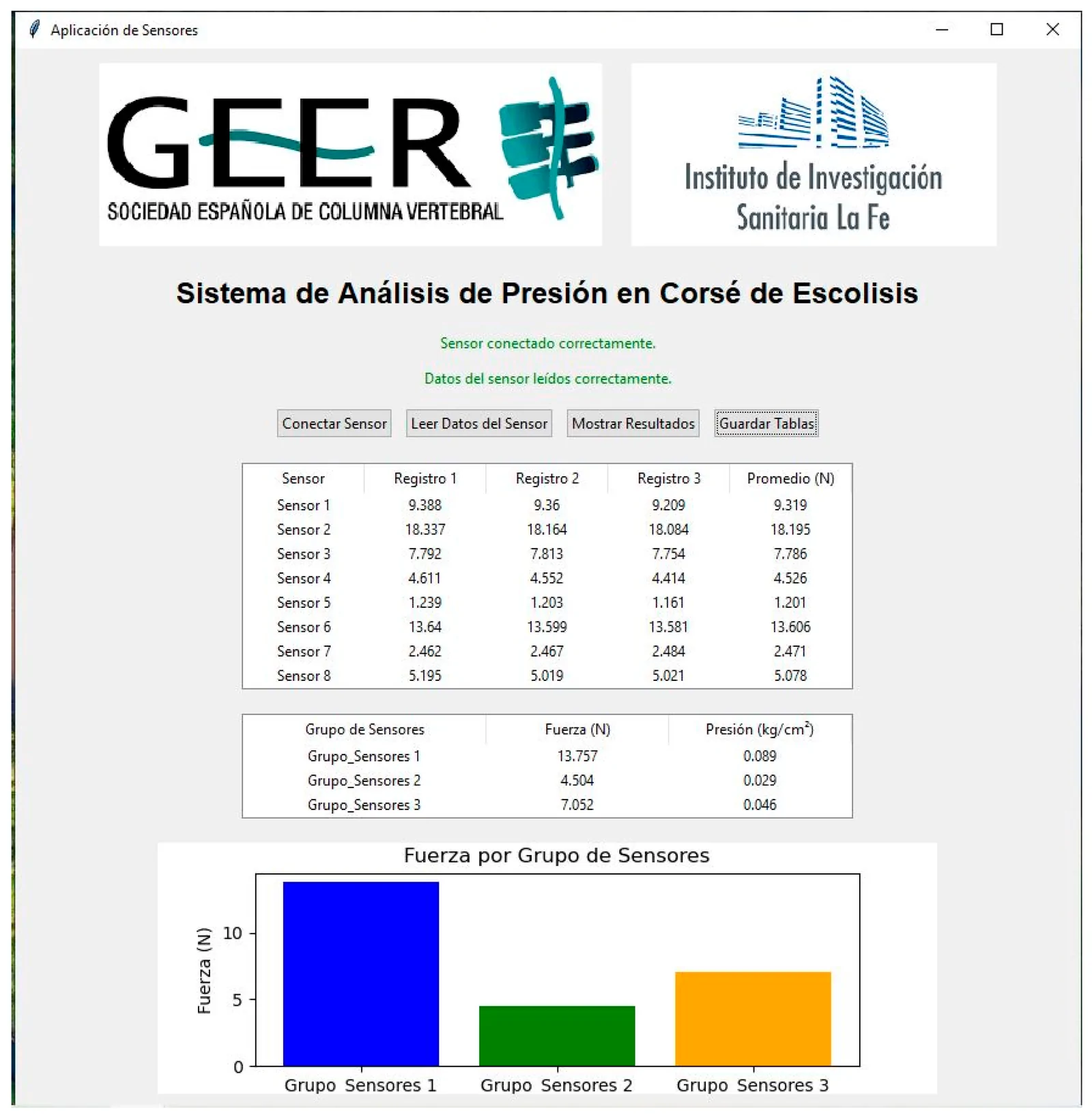
Screenshot obtained from the Spanish-language application designed for adjustment evaluation. Entitled *System of Pressure Analysis of Scoliotic Brace*, it presents an interface that allows the user to connect the sensor, read the data, display the results, and save the results table. In addition, it provides a graphical representation of the force, in Newtons, recorded by each group of sensors.

Data from the temperature and pressure sensing system are collected from two sources:

1. Adherence Monitoring: Using the Tucky^®^ app, once connected, the sensor continuously measures body temperature. Data are stored on the mobile device and synced with a secure online platform (Figure 5). Clinicians can log in, define a date range, and export records in Excel format.

2. Brace Fit Monitoring: A custom application was developed for pressure readings. For each reading, the clinician performs 10 consecutive measurements, stored in a matrix. The system then displays processed data, calculates averages per sensor and region (thoracic, lumbar, pelvic), and presents the results in two tables (individual records and cumulative values) and a bar graph (Figure 6).

### 3.5. Patient Pilot Testing

A total of four patients (n = 4, mean age 12.5 years, 100% female) were included. Pressure parameters are presented in Table 2.

## 4. Discussion

This study aims to design and develop a cost-effective, efficient prototype capable of objectively measuring both adherence and brace fit in patients with adolescent idiopathic scoliosis (AIS). This device, named *Cor-Esc-25*, is the first step toward a future device which will address this need. The results demonstrate that the combined use of low-cost pressure and temperature sensors could provide data enabling a comprehensive understanding of both patient behavior regarding brace use and the brace’s mechanical action on the patient.

### 4.1. Validation of the Temperature Sensor

Although some studies assess brace adherence using pressure-based systems, most groups prefer temperature sensors, as pressure readings may fail in cases of loose orthotic fit [5,6,7,9,12]. Rahman et al. used the Cricket^®^ sensor, a compact temperature logger, and concluded that it provided significantly more accurate adherence data than patient self-reporting. However, the authors noted that battery drain and lack of digital memory limited its results [11].

Brace usage is typically lowest during school hours and highest during nighttime [5]. The Tucky^®^ sensor has a battery life of up to five days, and the authors propose recharging it while the child is at school. Regarding data storage, the system allows real-time synchronization with a cloud platform, meaning that memory capacity is not a limiting factor.

On the other hand, Zou et al. support the use of a combined pressure and temperature system to monitor adherence. They argue that using only a temperature sensor may be ineffective in hot climates exceeding 28.0–33.0 °C [5]. However, the costs associated with a dual system exceed the budget considered in this study. Although the Tucky^®^ sensor was not specifically designed for use in a brace, the data it provides are very similar to those reported in the literature [5,6,7,9,12]. Therefore, we consider it to be a valid and reliable option for adherence monitoring in conservative AIS treatment. However, real-life studies are needed, especially in warm climates to enhance the sensibility of the sensor in in vivo conditions.

### 4.2. Validation of the Pressure Sensor

The first study to investigate pressure within a brace was conducted by Wong and Evans in 1998, using an electrohydraulic system that recorded values of 0.0093 ± 0.0013 MPa [24]. More recently, Ahmad et al. used the F-Socket system to measure the interface between the Chêneau brace and the patient, reporting values of 0.067 ± 0.013 MPa [25]. The F-Socket system is specifically designed for pressure measurement in orthotic devices and was considered in the market analysis phase of our study. However, it was discarded due to its high cost (EUR 25,549) [21].

Interestingly, the pressure values reported by Ahmad et al. are up to 100 times higher than those recorded by Wong and Evans (Milwaukee brace: 0.0093 ± 0.0013 MPa), Pharm et al. (Chêneau brace: 0.0073 ± 0.00064 MPa), and Fuss et al. (Chêneau brace: 0.056 ± 0.029 MPa) [8,24,26]. Van den Hout et al. reported dual measurements in the Boston brace, recording mean values of 0.043 ± 0.005 MPa at the thoracic level and 0.015 ± 0.004 MPa at the lumbar level [27].

These discrepancies may be attributed to (1) the different types of braces used (Boston, Chêneau, Milwaukee), and (2) the variety of pressure measurement systems employed across studies, which span more than 20 years. During this time, both sensor technology and clinical guidelines for strap tension likely evolved.

Nonetheless, the pressure values obtained using our custom-designed sensor fall within the range described in recent studies: 0.018 ± 0.004 MPa for Sensor 1 (thoracic curve), 0.00184 ± 0.008 MPa for Sensor 2 (lumbar curve), and 0.022 ± 0.008 MPa for Sensor 3 (pelvis). These values are lower than the values reported in the literature This variability could be attributed to differences in measurement protocols, brace types, or patient-specific factors, including the placement of our sensors and the characteristics of the sensor surface used. However, we highlight the importance of future studies in which the *Cor-Esc-25* prototype could be used to study the variability of these values in the long term and determine if pressure changes are related to the correction of the Cobb curve.

Except for Van den Hout et al., most studies only measure interface pressure at a single point. Even Van den Hout et al. only included two points (thoracic and lumbar), whereas our system considers three clinically relevant contact points [27]. Tymińska et al. aimed to validate the use of graphene-based sensors by using multiple brace contact points, but their results focused on force (Newtons) rather than pressure (Pascals), making direct comparison with other studies impossible [14].

### 4.3. Final Design of Cor-Esc-25

The *Cor-Esc-25* was designed as an affordable, reproducible prototype capable of integration into any brace model to evaluate adherence and pressure measurements. While some studies have fully embedded sensors into the brace structure, in our design, only the adherence sensors are fixed to the brace, whereas the pressure sensors are to be added during each clinical visit [8,11,14].

Existing commercial systems typically measure either adherence or pressure—but not both. Even Zou et al., who implemented both types of sensors, focused only on adherence and not on brace fit [5]. Moreover, the addition of more electronic sensors increases the risk of issues related to battery life and data storage [11]. For this reason, we believe that integrating only the essential sensors into the brace will facilitate clinical implementation.

Another key design objective was to ensure a low production cost, supporting future large-scale clinical implementation. The estimated manufacturing cost—including sensors, electronics, and assembly—is under EUR 150 (*EUR 69.90* for the Tucky^®^ sensor and *EUR 50* for the pressure system). This represents only a fraction of the cost of other commercial systems previously cited [8,11,14,25]. This affordability, without compromising basic measurement reliability, positions *Cor-Esc-25* as a viable alternative for hospitals, research institutions, and orthotic manufacturers.

### 4.4. Limitations

This study has several limitations. First, temperature measurements were performed in controlled conditions and not systematically tested in AIS patients, whereas pressure measurements were tested in four AIS patients only. Although we suggest the potential validity of our device, the data do not yet reflect in vivo conditions and do not yet have clinical correlation. Furthermore, validation was performed under static conditions, not during physical activity or daily movements, which could affect the alignment and accuracy of the Tucky^®^ sensor.

The Tucky^®^ sensor requires a dedicated mobile application and Bluetooth connection within a range of 10–15 m. Although most adolescents own a smartphone, this must be assessed on a case-by-case basis. Additionally, the sensor requires recharging every five days, potentially resulting in data gaps. Reliance on patient-owned smartphones and consistent Bluetooth pairing could introduce significant user burden and potential adherence monitoring failure. The Tucky^®^ sensor has only been tested in in vitro conditions, and temperature in warm climates could influence its results. Its placement in a ventilation hole could alter measurements.

While our system includes three pressure sensors—a step forward compared to previous studies—it may still not capture all relevant pressure variations across the brace.

The *Cor-Esc-25* project has been approved by our institutional ethics committee for use in AIS patients, and most of the aforementioned limitations will be addressed in subsequent clinical studies.

## 5. Conclusions

*Cor-Esc-25* is a low-cost, adaptable prototype designed to objectively monitor both brace adherence and fit in AIS. In this proof-of-concept study, feasibility was demonstrated through a pilot trial in four AIS patients, showing that the system can reliably acquire temperature- and pressure-based data.

Although the current results are preliminary and limited to static conditions, *Cor-Esc-25* represents a promising step toward affordable, integrated monitoring systems. Future work will focus on developing a second-generation device with full sensor integration, improved usability, and large-scale clinical validation.

## Figures and Tables

**Table 1:** Qualitative summary of the sensors selected in the market study.

Product	Price	Specific for Brace	Connection	Memory	Battery
ScolioSense^®^	-	Yes	Bluetooth	-	21 days
Orthotimer^®^	*EUR 2646*	Yes	Bluetooth	Internal (400 days)	4–5 months
Boston Sensor^®^	-	Yes	Bluetooth	Online platform	1 year
Core^®^	*USD 298.95*	No	Bluetooth and ANT+	Online platform	6 days
Radius T^®^ Masimo	*USD 49.99*	No	Bluetooth	Online platform	8 days
Tucky Sensor^®^	*EUR 69.90*	No	Bluetooth	Online platform	5 days

**Table 2:** Summary of pressure parameters recorded by each sensor in four selected patients.

Site	Mean Pressure (MPa)	SD	Range
Sensor 1 (Thoracic)	0.018	0.0043	0.011–0.022
Sensor 2 (Lumbar)	0.0184	0.0082	0.006–0.029
Sensor 3 (Pelvic)	0.0222	0.0084	0.02–0.039

## Data Availability

The original contributions presented in this study are included in the article. Further inquiries can be directed to the corresponding author.

## Abbreviations

The following abbreviation is used in this manuscript:

AIS	Adolescent Idiopathic Scoliosis
